# Software engineering for scientific big data analysis

**DOI:** 10.1093/gigascience/giz054

**Published:** 2019-05-23

**Authors:** Björn A Grüning, Samuel Lampa, Marc Vaudel, Daniel Blankenberg

**Affiliations:** 1 Bioinformatics Group, Department of Computer Science, University of Freiburg, Georges-Koehler-Allee 106, D-79110 Freiburg, Germany; 2 Center for Biological Systems Analysis (ZBSA), University of Freiburg, Habsburgerstr. 49, D-79104 Freiburg, Germany; 3 Pharmaceutical Bioinformatics group, Department of Pharmaceutical Biosciences, Uppsala University, Box 591, 751 24, Uppsala, Sweden; 4 Department of Biochemistry and Biophysics, National Bioinformatics Infrastructure Sweden, Science for Life Laboratory, Stockholm University, Svante Arrhenius vag 16C, 106 91, Solna, Sweden; 5 K.G. Jebsen Center for Diabetes Research, Department of Clinical Science, University of Bergen, Postboks 7804, 5020, Bergen, Norway; 6 Center for Medical Genetics and Molecular Medicine, Haukeland University Hospital, Postboks 7804, 5020, Bergen, Norway; 7 Genomic Medicine Institute, Lerner Research Institute, Cleveland Clinic, 9500 Euclid Avenue / NE50, Cleveland, OH, USA

**Keywords:** software development, big data, workflow, standards, data analysis, coding, software engineering, scientific software, integration systems, computational tools

## Abstract

The increasing complexity of data and analysis methods has created an environment where scientists, who may not have formal training, are finding themselves playing the impromptu role of software engineer. While several resources are available for introducing scientists to the basics of programming, researchers have been left with little guidance on approaches needed to advance to the next level for the development of robust, large-scale data analysis tools that are amenable to integration into workflow management systems, tools, and frameworks. The integration into such workflow systems necessitates additional requirements on computational tools, such as adherence to standard conventions for robustness, data input, output, logging, and flow control. Here we provide a set of 10 guidelines to steer the creation of command-line computational tools that are usable, reliable, extensible, and in line with standards of modern coding practices.

## Background

Big data has emerged as an era-defining characteristic for modern science. The design, implementation, and execution of computational tools is critical to the understanding of large datasets. Increasing attention is being paid to various facets surrounding computational research, including reproducibility [1, 2], reusability [3, 4], and open source efforts and community building [5, 6]. Previous resources [7–9] have provided useful recommendations for beginning research software development and are recommended reading. However, a single set of guidelines for new (and veteran) scientists on software development for big data is still lacking. One area that is particularly underrepresented in the literature is the increasing need to incorporate scientific software tools into workflow management systems, tools, or frameworks that coordinate the execution of multiple such tools in complex dependency schemes. Integration into such workflow systems (e.g., [10–16]) adds its own layer of requirements on computational tools, such as following standard conventions for data input, output, and logging, and allowing workflow systems to override default behaviors in these areas when necessary.

As datasets become larger and more complex, and as computational analysis becomes more mainstream, research scientists may find themselves in the position of impromptu software engineers. These researchers will, at a minimum, need to learn about defining software requirements and designing, constructing, testing, and maintaining software. Unfortunately, there are few resources dedicated to helping a scientist transition from writing one-off scripts to developing computational tools that are usable by others and can easily be incorporated as components in pipelines and workflows consisting of many interoperating tools. As such, many of the computational tools released can only be described, politely, as “research-grade.” These tools can place an exorbitant burden on the end users who find themselves in the position of attempting to execute and chain them into complex analysis pipelines.

Here we provide a set of 10 guidelines to steer the creation of command-line computational tools that are usable, reliable, extensible, and in line with standards of modern coding practices. But before diving into these guidelines, it is necessary to highlight the difference between 2 different possible modes of development that can be useful in research: (i) prototype and (ii) production. Software prototypes can be highly beneficial in science as a way to explore the ins and outs of an application domain of interest, possibly before a software engineering effort starts and a production tool is developed. However, it is important to keep a clear distinction between prototype coding and the software engineering required to create a robust, reliable tool that is widely usable, and it is crucial to convey this distinction to users. Prototype coding is typically more explorative in nature and might lack normal reliability measures, such as tests, for maximizing development speed. Conversely, to create reliable software that is usable by others, the requirements for careful planning, specification, testing, and verification are vastly more stringent. The guidelines below address the development of the latter category: building well-engineered, reliable, robust, and reusable software with a clearly defined purpose. They are by no mean exhaustive but aim rather at highlighting factors that will be essential to the successful development of scientific software for big data.

It is worth noting that practical realities of research software development, such as limited funding and publication pressure, often favor the prototype modality. Simply put, research funding and scientific reward systems have historically discouraged the creation of production-quality software as being non-essential; if a set of code is able to perform the specific task necessary, under the specific environmental context, then it is good enough for inclusion within a publication and it is best to publish as quickly as possible. Given these systematic conditions, it is understandable that software quality and usability are often not pursued; however, it is a hindrance to scientific progress and, while we should demand better, at least the code is available. Furthermore, there is often the case that software begins as a prototype but then becomes production-oriented through a process of continuous improvement. These agile approaches can be powerful and effective processes for creating robust software that meets the evolving needs of a user base. This review is intended to discourage neither the creation of prototypes nor the use of agile software development practices, and the authors continue to utilize these approaches when advantageous to a project. However, these guidelines are helpful to keep in mind during initial prototyping and should be converged upon in cases when a prototype becomes production-quality distributed software.

## Guidelines

### Guideline 1: Failing to plan is planning to fail

Spend adequate time designing the scope and expectations of your project. A successful scientist does not simply run to the laboratory bench with a vague idea of an outcome and start mixing colorful liquids together with the hope of producing something useful. This same type of careful and mindful planning must be engaged in while developing computational tools. Spec out exactly what your tool needs to do. Define all use cases, input datasets and data types, configurable parameters, set of actions, and desired outputs and types. Anticipate what is beyond the current scope of your software-what use cases, data types, and configurations sound interesting but fall beyond the initial scope of your software. Sketching out the components and dataflows of the software on a whiteboard, or on paper, can be particularly helpful at this stage. Clearly define the scope of your work, and anticipate corner cases that you think you might need to account for; e.g., are you interested in a circular genome that is going to be represented as a linear sequence for input? Finally, speculate on the factors that will indicate that the software needs to be refactored, replaced, or retired.

Once all of the specifications are set, it is time to search, and search thoroughly, to see whether there is an open source tool with clear measures of reliability, such as automated software tests, that does what you want or something very similar. A good place to start is simply to google your initial question or navigate StackOverflow-it is likely that someone has already bumped into the same or a similar problem. If a reliable tool exists that does what you want, consider using it, even if it requires using several tools pipelined together. If there is a reliable tool that exists that does something close to what you need, consider modifying it. There is no need to design a brand-new blue bicycle if you can paint an existing white one and achieve the same goal. When assessing the quality of an existing tool, you should follow the same guidelines that are laid out here for designing your own software: (i) Can you understand what the code does, so that you can verify its logic and assumptions, can fix bugs, and improve the code if required? (ii) Does the software contain tests? (iii) Does it have a high test coverage? (iv) Does it use continuous integration? (v) What other dependencies does it rely on? (vi) Are those dependencies deemed robust, reliable software? (vii) Are the licensing and implementation compatible with integration in your workflow? For tools that lack the required reliability measures such as software tests, consider contributing these missing tests yourself, thereby helping both yourself and other potential users of that software. When doing this mining work, do not hesitate to contact the developers of other tools or users facing the same need as you. Interacting with the community will help to better define the project.

The reason for doing such careful and exhaustive research of existing software is that the cost of maintaining yet another piece of software is almost always vastly underestimated. Therefore, generally only consider developing new software if there are clear signs that you can lessen the maintenance burden by creating a drastically improved or simplified implementation that makes it easier to understand, test, and refactor the code, and that such improvements are not possible with the existing software itself. Making this determination is a difficult task, one that can be challenging for even the most seasoned software industry professionals, and in the end rests on what is essentially a judgment call that should consider all aspects, including not only the code, but also the existing developer and user communities, of the software under consideration. Additionally, although outside the scope of this review, there are several significant and valid alternative reasons for reimplementing code, such as learning exercises, to explore and validate expected or presumed algorithmic behavior, and so forth. Finally, should you decide to develop a new tool, there might be important lessons to be learned from, or parts of the code that can be reused from, existing open source tools. Once you have exhausted all existing options, it is time to move on to the next step.

### Guideline 2: Build test-driven trustworthy code

Instead of coding and then testing, approach from the other way around: before you can begin to convince others that your software implementation is correct, you need to prove it to yourself. Obtain or create a set of input files, and design a set of outputs that are expected to be created across various parameter settings. By relying on sets of known inputs and outputs designed from the planned requirements, you can be confident that the results generated by your software will match the intended requirements. Additionally, test-driven development forces coding efforts to concentrate on features that are needed according to the well-planned requirements, preventing feature creep. Looking at the metaphorical answer at the back of the book helps in steering development, thereby creating more precise code.

The “tests first” mantra should drive the development of every aspect from coding to deployment. From the very beginning to the very end, unit tests should be designed to test individual methods and classes while integration and functional testing should be used to confirm the overall correctness of the final application. Especially in the case of the development of new algorithms and approaches that are only exercised when subjected to real data, it may not be practical to design a full test suite to verify the complete correctness of the approach until after it has been mostly coded and executed. In these cases, it is still important to test as much as possible, as early as possible, with initial tests and then to be sure to establish a test-set that encompasses the entire behavior. A best practice for test suites is to start with a clean environment, install your software, execute your code, and then verify correctness. Testing may also include syntax checking, test coverage checking, checking against coding conventions (often called “linting”), and automatic memory leak detection. It is also good practice to provide negative tests, which will ensure that errors are properly handled. There are many tools that can help testing for virtually every programming language. Take advantage of existing test harnesses and continuous integration suites, such as Jenkins [17], Travis [18], and CircleCI [19].

### Guideline 3: Denominate mindfully and code for the future

Naming things is hard. When writing software, there are many things that will need to be named, such as functions, variables, classes, parameters, program arguments, and so on. Names must be self-explanatory to those who will be interacting with them. They should describe the intended purpose and not implementation details. While computers do not care, a method named “do_thing()” is not descriptive to an individual who may need to read, maintain, or modify the code later.

Names should be chosen very carefully-check for typographical errors-so that they will not need to be changed in the future, which could result in backwards incompatibility. You should avoid renaming user-exposed items as much as possible and especially forgo reusing or repurposing existing or previously used short or long argument names. For example, if an argument was accessible using “-o/$\hbox{-}\hbox{-}$orthogonalOption”, you should not decide later to use “-o” to specify the output filename. If it is necessary to break backwards compatibility, be sure to do so using well-defined semantic versioning, and by providing conspicuous and prominent notice to potential users, many of whom will not think of reading release notes (or even realize that they exist). It is also important to consider that changes to existing command-line arguments can greatly increase the burden on users when they desire to upgrade to a new version. For a simple case this may just require a user to remember to enter a new set of arguments, but, for workflows and other integration systems, where the easiest upgrades could otherwise be performed by only modifying a version string, required changes could be significantly more in depth, even when a pipeline is not modified to take advantage of new features.

While your code may seem perfectly clear at time of writing, things will be very different in 5-10 years-or simply a few days before an important deadline when everything breaks. Give space to your code; organize it so that it is compartmented, clear, and pleasant to read. Making your code understandable to all will help others to find bugs and implement features. It will also facilitate collaborative development and take some maintenance work off your shoulders.

### Guideline 4: Stick to standards

Stick to standards: standard file types, standard ontologies (e.g., EDAM [20]), standard command-line arguments and naming schemes ($\hbox{-}\hbox{-}$help/-h, $\hbox{-}\hbox{-}$version/-v), and do not create your own dialect or derivative file format. By utilizing standard file formats, a software tool is made inherently interoperable, allowing simplified use within existing analysis workflows. Adopting standard ontologies allows tools to be easily discoverable. At a minimum, you should provide $\hbox{-}\hbox{-}$version and $\hbox{-}\hbox{-}$help commands in every tool. The help should provide enough details for a typical user to be able to make use of the tool. Include a $\hbox{-}\hbox{-}$version command to enable reproducibility through capturing the version number of your tool easily. Try to avoid defining the version number at multiple locations within your code. Instead, design your code in such a way that the version definition is declared once and then referenced as needed. If possible, try to include the version control system revision number into the output of the version (sometool v1.2.3-5483e9f5bf4d725e3). Take advantage of semantic versioning [21] similar to MAJOR.MINOR.PATCH, where changes in PATCH indicate backwards compatible bug fixes, MINOR indicates backwards compatible feature additions, and MAJOR can indicate large changes that may not be backwards compatible.

Of particular importance is to take advantage of language-specific coding standards, as any deviation will make future maintenance much more difficult. For example, in Python, this can mean using indentation of 4 spaces, not 3, not 5, not tabs, and certainly not mixed. In Perl, this can mean using parentheses for readability even when not strictly required. When using scripting languages, the shebang (#!) is a powerful single line of code, but you should use it correctly. Use "/usr/bin/env python" or "/usr/bin/env perl" instead of "/usr/bin/perl." It is not guaranteed that Perl is installed in "/usr/bin/," especially when using specific versions of a software program in virtual environments, such as those created by Conda [22]. In all cases, make proper use of exit codes to report the terminal status of your tool; a 0 means everything in the world is good, and anything other than a 0 is an error.

Make use of previously existing stable libraries and packages (e.g., Biopython [23], pysam [24], BioPerl [25], SeqAn [26], BioJava [27], BioJS [28]) whenever possible, including the specific version of each required dependency in the installation methodology. Beware of licensing conflicts. For libraries and packages that are not available in stable repositories or standard distribution channels such as those mentioned above, but, e.g., only in a single developer's git repository, it is recommended to ensure the availability of the library or package in some way, such as by submitting the package to a stable repository or distribution channel, making a fork of the library into a repository that you as developer have control over, or additional backup method. Be sure to include an accepted standard open source license with your code. Adopting a customized or oddball license can lead to issues downstream and greatly hinder community acceptance. The Open Source Initiative, a non-profit organization that promotes and protects open source software, projects, and communities, provides guidance on selecting from a list of approved open source licenses [29].

Adopt well-established standard frameworks and approaches to handle common problems. For example, if you have an easily parallelizable problem, e.g., if your tool consumes a multiple FASTA file and operates on each unit independently, then it is a good idea to split the FASTA file into small chunks to speed up your tool by using multiple processing cores. However, do not reinvent the wheel when adding these optimizations, instead make use of existing reliable tools and frameworks; in particular, become well-versed in common GNU/Linux utilities such as awk, grep, split, parallel, etc., to handle large files.

### Guideline 5: Choose sane defaults but allow overriding

When providing default values for parameters, choose settings that make the most sense for the typical application of the tool. In the help for your tool provide the default value used, and the effects that changing it will have. Allow default values to be overridden by environmental variables and by supplying a command-line argument, in increasing order of preference.

For example, when creating temporary intermediate files, use predefined libraries from your favorite programming language (like tempfile in Python), which will place files in the location defined using operating system-defined mechanisms. This enables the cluster admin to have the option to assert the use of fast local hard drives for temporary storage, which can drastically speed up input/output-heavy computational tasks. While the temporary directory is often controllable by using an environment variable ($TMPDIR), it can also be useful to allow this to be changed using a tool-specific environment setting (e.g., $MY_TOOL_TEMP_DIR) and by passing an explicit argument to the tool (e.g., $\hbox{-}\hbox{-}$tmpdir). This gives the system administrator, users, and workflow management systems multiple injection points, resulting in the greatest flexibility to control data flow. When there are multiple ways to specify a parameter value, log the final value utilized to the standard application log when the tool starts, so that it is easy to spot any values being overridden by accident. At completion, log all values and assumptions used for traceability and reproducibility of the results.

### Guideline 6: Make no assumptions

“Never assume, because when you assume, you…” Assumptions can result in cryptic errors, or worse, erroneous results that are reported without notification. Do not assume that the user will have a home directory for storing files and configurations (e.g., .programNameConfig). In many cluster environments, jobs and tasks may not have access to a home directory. And they almost certainly do not have *your* home directory, so please do not hardcode that. As a general rule, you will be surprised by how creative your users will be at using your code and breaking it. If something can go wrong, it will- programming with Murphy's Law in mind will save you countless hours of debugging.

Do not rely solely on file extensions to determine the file type. As a default setting this is good, but offer alternative ways to instruct your program which file type is provided. File extensions are often not standardized. Moreover, data management systems might store the file type and other metadata in a database separate from the dataset contents, without a file extension or human-readable filename. Often these datasets are identified by a hashsum or a universally unique identifier. Enabling compatibility with object-based storage is a worthwhile goal. Similarly, if you define a novel file format, make an effort to give automatic type detection systems the ability to identify files, such as by making use of magic numbers or declarative headers. Data management systems might also need to use a modified filename while a file is being created, to enable the separation of finished and unfinished files, such as in the case of a crashed workflow run, so that unfinished files are not erroneously reused when the workflow is restarted.

Ideally, allow the ability to customize completely the filename of every input file and associated metadata file, and every output file and associated metadata file generated by the tool. For example, it is bad practice to only allow the tool to accept a file named input.bam that is located in the current working directory. If, for any reason, the tool cannot reasonably take the exact filename for every output file (e.g., when the number of outputs is unknown or very large), allow the user to specify both an output directory and a filename pattern.

### Guideline 7: Be one with the streams

You can see every tool as a filter: it turns an input into an output. Whenever possible, make your tool streaming aware. Filesystem input/output is increasingly becoming a bottleneck in modern computing. Allow the primary input and output datasets to be consumed and produced as streams, which are only temporarily stored in random-access memory (RAM), so that they can be used effectively with redirection, and, more importantly, pipes (|), preventing the need to read and write from disk when the tool is used as part of a multi-tool process. When working with streams, be aware that for input and output, text is often king. Furthermore, the use of standard stream-aware GNU/Linux utilities, which primarily operate in the text-based realm, can be mightily powerful for processing and filtering.

### Guideline 8: Metadata is valuable

Metadata is as important as the primary dataset. Try to stick to existing standard file formats, but if it is necessary to create a new or extend an existing file format, be sure to make provisions for storing metadata. Include enough information to unambiguously identify the file type, version, and meaning behind values (e.g., column names). It is a good practice to also include information about the generating program, including version and complete set of parameters. Do what you can to keep data and its associated metadata as closely attached as possible, and in sync. If at all possible, avoid using metadata files (file.ext and file.ext.ext2), but include metadata within a metadata section or header of the dataset. This ensures that data and metadata remain in sync and do not accidentally diverge, e.g., by accidentally moving a subset of the files around on a file system. If separate metadata files need to be created, it can in some cases be helpful to package data and metadata together in an archive file or container format such as tar, zip, or HDF5.

### Guideline 9: Users come first

Computational tools must be usable by the widest range of users. The tool should be executable on user-provided data without requiring them to jump through numerous steps that could be easily automated. One common example of this is requiring input datasets and metadata to use a particular filename or follow a specific pattern. The user should be able to provide filenames directly and be able to make use of standard command-line interpreter features and shellisms such as wildcards and tab-completion. The use of a file to specify input parameters, instead of accepting command-line arguments, is also discouraged because it unnecessarily complicates the notion of input files by creating 2 different classes of input files. Making it easy to run your tools will be rewarded by increased adoption by the community and decreased support and maintenance burden.

Installation of the computational tool is likewise important; installation procedures for a tool and its dependencies can become a challenge for users of different backgrounds [30]. Simply put, if a tool cannot be installed, it cannot be used. Try to make use of the default installation mechanism of the programming language. There are many tools that do not obey these rules and require patching and additional scripting in order to be installed. Do not include compiled binaries or external source code within your version control system. Binaries should be either generated from the code base or provided by a package management system. External source code should be resolved with the installation mechanism (e.g., GNU Autotools [31], pip [32], Pom/Maven [33]). The use of Conda, particularly conda-forge [34] and Bioconda [35], has been shown to be very effective in providing versioned ready-to-go cross-platform tool environments. Finally, mind the system requirements of servers handling sensitive data: they generally offer limited connection to the Internet and do not allow running all kinds of containers.

### Guideline 10: Documentation is paramount

Providing good end user documentation is essential to enable users to effectively use any computation tool. Simplified, succinct documentation should be provided onscreen with a $\hbox{-}\hbox{-}$help argument to the tool. More in-depth documentation should be provided via manual pages, web pages, and PDF documents. Providing tutorials, or vignettes, that walk through typical use cases is particularly beneficial to users. A good approach is to embed the documentation within a “doc” directory of the source code in a human-readable language such as Markdown. These Markdown files can then be automatically converted into stylized HTML or PDF documents. It is a good practice to provide clear support avenues such as a mailing list, forum, and issue tracker where users can request assistance and report bugs. These services are greatly simplified by open source public code repositories, such as GitHub [36], GitLab [37], and Bitbucket [38]. Finally, consider including a contributing document that informs other community developers how they can commit code or otherwise help with your project.

Take advantage of the version control system to transparently document every change in the code. The version control system is as important when developing software as a lab book during an experiment. Provide an accurate but concise change log between versions. This change log should be easily understandable by the end user, and highlight changes of importance, including changes in behavior and default settings, deprecated and new parameters, etc. It should not include changes that, while important, do not affect the end user, such as internal architectural changes. In other words, this is not simply the unannotated output of "git log."

## Conclusions

Progressing from writing simple one-off scripts to developing truly useful and reusable computational tools requires substantially more planning and increased considerations. Do not rush through the initial steps. Time spent designing the scope and expectations of a project, including input datasets and formats, configurable parameters, and desired outputs and output types is time well spent. By starting with a set of tests, you can ensure that your tool is functionally correct. Adopt and use existing, well-established, tools, file formats, and other standards whenever they are available. Do not make changes that will break backwards compatibility, except when absolutely necessary, and increment software versions appropriately. Be sure to choose sane default values for tool parameters, but allow users to easily change their values. Do not make any assumptions about the local compute environment or infrastructure. Making your software stream-aware goes a long way towards encouraging its use on high-performance computing resources and incorporation into existing computational pipelines. Metadata is as important as the primary datasets; be sure to treat it with the same care, and try to keep it as closely connected to the primary data as possible. Always be aware of the various types of users that your software will have, and tailor your tool, development approaches, and documentation to support them. As you continue to advance from a simple script writer to a software developer, do not unnecessarily fret about unknown bugs or deficiencies in coding ability, knowledge, or style, because everyone starts somewhere, but be sure to avoid developing a bad code-ego [39], keep learning about open source best practices (e.g., [40, 41]), and do not hesitate to reach out to the community.

## Competing interests

The authors declare that they have no competing interests.

## Funding

Supported with funds provided by the Cleveland Clinic, and the European Union's Horizon 2020 research and innovation programme under grant agreement No 654241 for the PhenoMeNal project.

## Authors’ contributions

All authors wrote the manuscript. All authors read and approved the final manuscript.

## Supplementary Material

giz054_GIGA-D-18-00313_Original_Submission

giz054_GIGA-D-18-00313_Revision_1

giz054_GIGA-D-18-00313_Revision_2

giz054_Response_to_Reviewer_Comments_Original_Submission

giz054_Response_to_Reviewer_Comments_Revision_1

giz054_Reviewer_1_Report_Original_Submission -- Janna Hastings 9/4/2018 Reviewed

giz054_Reviewer_2_Report_Original_Submission -- C. Titus Brown 10/21/2018 Reviewed

giz054_Reviewer_2_Report_Revision_1 -- C. Titus Brown 2/5/2019 Reviewed
