# Supplementary material for: Software engineering for scientific big data analysis
Source: Gigascience. 2019 May 23;8(5):giz054. doi: 10.1093/gigascience/giz054 (PMC6532757; doi:10.1093/gigascience/giz054)
Supplement: giz054_GIGA-D-18-00313_Revision_1 [file giz054_giga-d-18-00313_revision_1.pdf]

GigaScience

Software engineering for scientific big data analysis

--Manuscript Draft--

**Manuscript Number:**	GIGA-D-18-00313R1
**Full Title:**	Software engineering for scientific big data analysis
**Article Type:**	Review
**Funding Information:**	Cleveland Clinic Foundation (Seed Funds) — Dr. Daniel Blankenberg<br>Horizon 2020 (654241) — Not applicable
**Abstract:**	The increasing complexity of data and analysis methods has created an environment where scientists, who may not have formal training, are finding themselves in the impromptu role of a software engineer. While several resources are available for introducing scientists to the basics of programming, researchers have been left with little guidance on approaches needed to advance to the next level for the development of robust, large-scale, data analysis tools that are amiable for integration into workflow management systems, tools, and frameworks. The integration into such workflow systems necessitates additional requirements on computational tools, such as adherence to standard conventions for robustness, data input, output, logging, and flow control. Here we provide a set of ten guidelines to steer the creation of command-line computational tools that are usable, reliable, extensible, and in line with standards of modern coding practices.
**Corresponding Author:**	Daniel Blankenberg<br>Cleveland Clinic Lerner Research Institute<br>UNITED STATES
**Corresponding Author Secondary Information:**	
**Corresponding Author's Institution:**	Cleveland Clinic Lerner Research Institute
**Corresponding Author's Secondary Institution:**	
**First Author:**	Björn A. Grüning
**First Author Secondary Information:**	
**Order of Authors:**	Björn A. Grüning<br>Samuel Lampa<br>Marc Vaudel<br>Daniel Blankenberg
**Order of Authors Secondary Information:**	
**Response to Reviewers:**	PDF also attached.<br><br>Dear Scott,<br><br>We want to take this opportunity to thank you and the referees for reviewing this manuscript. We are greatly appreciative of the time and effort expended and feel that the provided comments, suggestions, and queries have resulted in an improved submission.<br><br>We have replied to the reviewers’ comments inline below, and have made referenced changes in the submitted revision.<br><br>Please do not hesitate to contact me if there are additional comments or concerns.

Powered by Editorial Manager® and ProduXion Manager® from Aries Systems Corporation

Sincerely,

Daniel Blankenberg
blanked2@ccf.org
Office: 216-445-4336
Cell: 216-212-2211

Reviewer #1: This is a nice, neat article about best practices in software engineering as applied to the development of command-line utilities for scientific data processing. In general, there is little with which to disagree in the article. However, I would recommend the authors consider a few changes to clarify the central message. Firstly, although the title says "Software engineering for scientific big-data analysis", there was little in the guidelines presented that seemed to me to be specific to big-data analysis. The authors might consider highlighting which aspects of the software engineering guidelines they present are uniquely relevant to big data.

This is a good point. In fact, we would agree that the guidelines presented here are applicable and helpful for software engineering in general. However, the nature of big data analysis with increased concerns such as dataset and workflow/pipeline complexity, availability and costs of computational resources, etc., necessitates increased vigilance. The cost of having inadequate or erroneous software in big data analysis is amplified while, in many cases, becoming simultaneously more difficult to uncover. It is our goal to raise awareness and address these concerns under this context.

Secondly, the authors distinguish at an early stage between software prototyping vs. production. However, in research contexts it is particularly commonly the case that the prototype *becomes* the production software in an evolutionary fashion, without sufficient time or funding to pause to consider how to engineer a production version. Of course, this is not because individual researchers necessarily lack the expertise to develop production-level software. Rather, it reflects the broader, environmental system of research funding and reward schemas, which are often outside of the control of individual researchers. I wonder if it would be useful to discuss this issue, given the central focus of your article on the processes involved in careful production-level processes.

We have added an additional paragraph to the background section discussing the usefulness of prototypes, the common case when a prototype converges into a distributed software package, and the realities of creating production-quality software under current funding and research-effort reward paradigms.

Reviewer #2: In this paper, Gruning et al provide their thoughts on "Software engineering
for scientific big-data analysis." It covers a variety of strategies to
produce reliable software for scientific data analysis at scale.

This was a tough paper to review. About half the time I found myself
cheering, about half the time I disagreed with some details, and about
half the time I found myself wondering why the authors were making this
point. (Yes, that adds up to 1.5 of the time.)

I'll start with my major disagreements --

There seems to be no allowance int his paper for the view that you can
produce robust software while coding your way to a better
understanding of the problem. I would term this an "agile" approach,
and it's something my group does regularly; it seems to work pretty
well and reaches the same end point these authors are suggesting, of
reliable and robust software that can be operated at scale. It just
does so circuitously. I don't even think the authors would disagree with

Powered by Editorial Manager® and ProduXion Manager® from Aries Systems Corporation

it. But it is simply not discussed in this paper.

We have added an additional paragraph to the background section discussing the usefulness of prototypes, the common case when a prototype converges into a distributed software package, and the realities of creating production-quality software under current funding and research-effort reward paradigms.

I think in general that this paper veers wildly between high level statements (robust software is important! create reliable software! plan ahead!) and minutia (use THESE command line options). Fine, but we're missing the middle view. I'm really not sure how to fix this, so I'll just leave it as an opinion for them to respond to!

This is a valid concern. The intention of this manuscript is to provide an easily consumable list of guidelines, along with illustrative examples. This is a hard balance to strike, especially when considering the open-endedness of potential programming languages, a variety of software development paradigms, text length and conciseness, etc. If there are specific items of particular concern, we would be happy to address them.

The authors laud reuse (but I'm curious how often they've been able to reuse existing software and adapt it - we virtually never can?) and ignore the *significant* value of reimplementing solutions from scratch, where you can detect implementation oddities that went undescribed in the initial paper. These tend not to be published directly, but you can look at (e.g.) the Oyster River Protocol meta-analysis paper by MacManes to see how undescribed algorithmic and parameter choices can have profound downstream impacts on outcomes. The other downside of reuse that is not really mentioned is the substantial complexity of so-called "general purpose" libraries; we don't use BioPython in my lab because we don't need 99% of it, and don't want to have to debug it.

These are excellent points. We completely agree that there are often times when reuse is not the best course of action, and in guideline 1 we present details on what to look for when judging whether or not to reuse a specific set of software.

These requirements set a relatively high bar for reuse. So, while we laud reuse and assert that it should be the default first approach, there are many occasions where it may not be prudent. For example, in the case of a many-domain general purpose library, it may not pass several of these requirements, including test coverage, understandability of code and ease of debugging, etc.

Addressing the specific case of the impacts of undescribed algorithmic and parameter choices of existing code can also be addressed by test coverage (having the correct set of tests based upon intended outcome), code clarity (being able to see implementation oddities), etc.

However, there are important reasons to reimplement code and we do not mean to imply that there is no value in it. We have added the following text to convey this sentiment: “Although outside of the scope of this manuscript, there are several significant and valid alternative reasons for reimplementing code, such as learning exercises, to explore and validate expected or presumed algorithmic behavior, and so forth.”

In some sense, a lot of this is covered in Morgan Taschuk and Greg Wilson's piece on making research software robust. Maybe the authors

	could focus on what they DON'T cover?<br><br>Morgan Taschuk and Greg Wilson provide a valuable resource with their paper, and we very early-on recommend its reading. Unfortunately, some overlap is unavoidable in order for this manuscript to stand on its own and to best service the reader; or, perhaps, it is fortunate, as the overlaps demonstrates importance and, potentially, aids with retention and adoption through multiple exposure. We view this manuscript as complementary to previous efforts, with each contributing individually, in addition to a common core.<br><br>Minor issues --<br><br>there's no guidance on choosing a license, at all. At the very least some citations would probably be a good idea. And please mention OSI licensing; there are a list of approved open source licenses to use, period.<br><br>We completely agree that the importance of carefully selecting a license is far too often undervalued or misunderstood. We added additional information about open source licensing to guideline 4 (new in bold): “Be sure to include an accepted standard open-source LICENSE with your code. Adopting a customized or oddball license can lead to issues downstream and greatly hinder community acceptance. The Open Source Initiative (OSI), a non-profit organization that promotes and protects open source software, projects and communities, provides guidance on selecting from a list of approved open source licenses (https://opensource.org/licenses).”<br><br>The statement "use containers" is somewhat undermined by "sometimes you can't".<br><br>Yes. This is true in the case of containers, and also true for the case of many other concerns in software. The intricacies involved, and a myriad of possible design choices, in building software make declaring steadfast rules difficult, which is why we present our recommendations as guidelines.
**Additional Information:**	
**Question**	**Response**
Are you submitting this manuscript to a special series or article collection?	No
**Experimental design and statistics**<br><br>Full details of the experimental design and statistical methods used should be given in the Methods section, as detailed in our Minimum Standards Reporting Checklist. Information essential to interpreting the data presented should be made available in the figure legends.<br><br>Have you included all the information requested in your manuscript?	Yes
**Resources**	Yes

Powered by Editorial Manager® and ProduXion Manager® from Aries Systems Corporation

A description of all resources used, including antibodies, cell lines, animals and software tools, with enough information to allow them to be uniquely identified, should be included in the Methods section. Authors are strongly encouraged to cite Research Resource Identifiers (RRIDs) for antibodies, model organisms and tools, where possible.<br><br>Have you included the information requested as detailed in our Minimum Standards Reporting Checklist?	
**Availability of data and materials**<br><br>All datasets and code on which the conclusions of the paper rely must be either included in your submission or deposited in publicly available repositories (where available and ethically appropriate), referencing such data using a unique identifier in the references and in the “Availability of Data and Materials” section of your manuscript.<br><br>Have you have met the above requirement as detailed in our Minimum Standards Reporting Checklist?	Yes

Powered by Editorial Manager® and ProduXion Manager® from Aries Systems Corporation

Manuscript

Click here to access/download;Manuscript;manuscript_coding_for_big_data_2

Click here to view linked References

Software engineering for scientific big data analysis

Björn A. Grüning[1,2,*], Samuel Lampa[3,4], Marc Vaudel[5,6], and Daniel Blankenberg[7,*]

[1]Bioinformatics Group, Department of Computer Science, University of Freiburg, Georges-Koehler-Allee 106, D-79110 Freiburg, Germany

[2]Center for Biological Systems Analysis (ZBSA), University of Freiburg, Habsburgerstr. 49, D-79104 Freiburg, Germany

[3]Pharmaceutical Bioinformatics group, Department of Pharmaceutical Biosciences, Uppsala University, Uppsala, Sweden (email: samuel.lampa@farmbio.uu.se)

[4]Department of Biochemistry and Biophysics, National Bioinformatics Infrastructure Sweden, Science for Life Laboratory, Stockholm University, Sweden

[5]K.G. Jebsen Center for Diabetes Research, Department of Clinical Science, University of Bergen, Norway

[6]Center for Medical Genetics and Molecular Medicine, Haukeland University Hospital, Bergen, Norway

[7]Genomic Medicine Institute, Lerner Research Institute, Cleveland Clinic, Cleveland, OH, USA; email: blanked2@ccf.org

[*]Co-first authors

Abstract

The increasing complexity of data and analysis methods has created an environment where scientists, who may not have formal training, are finding themselves in the impromptu role of a software engineer. While several resources are available for introducing scientists to the basics of programming, researchers have been left with little guidance on approaches needed to advance to the next level for the development of robust, large-scale, data analysis tools that are amiable for integration into workflow management systems, tools, and frameworks. The integration into such workflow systems necessitates additional requirements on computational tools, such as adherence to standard conventions for robustness, data input, output, logging, and flow control. Here we provide a set of ten guidelines to steer the creation of command-line computational tools that are usable, reliable, extensible, and in line with standards of modern coding practices.

Background

Big data has emerged as an era-defining characteristic for modern science. The design, implementation, and execution of computational tools is critical to the understanding of large datasets. Increasing attention is being paid to various facets surrounding computational research, including reproducibility [1,2], reusability [3,4], and open-source efforts and community building [5,6]. Previous resources [7–9] have provided useful recommendations for beginning research software development and are recommended reading. However, a single set of guidelines for new (and veteran) scientists on software development for big data is still lacking. One area that is particularly underrepresented in the literature is the increasing need to

incorporate scientific software tools into workflow management systems, tools or frameworks (SWMFs), that coordinate the execution of multiple such tools in complex dependency schemes. Integration into such workflow systems (e.g., [10–16]) add its own layer of requirements on computational tools, such as following standard conventions for data input, output and logging, and allowing workflow systems to override default behaviors in these areas when necessary.

As datasets become larger and more complex, and as computational analysis becomes more mainstream, research scientists may find themselves in the position of impromptu software engineers. These researchers will, at a minimum, need to learn about defining software requirements, designing, constructing, testing, and maintaining software. Unfortunately, there are few resources dedicated to helping a scientist transition from writing one-off scripts to developing computational tools that are usable by others, and can easily be incorporated as components in pipelines and workflows consisting of many interoperating tools. As such, many of the computational tools released can only be described, politely, as 'research-grade'. These tools place an exorbitant burden on the end-users that find themselves in the position of attempting to execute and chain them into complex analysis pipelines.

Here we provide a set of ten guidelines to steer the creation of command-line computational tools that are usable, reliable, extensible, and in-line with standards of modern coding practices. But before diving into these guidelines, it is necessary to highlight the difference between two different possible modes of development that can be useful in research: (1) Prototype and (2) Production. Software prototypes can be highly beneficial in science as a way to explore the ins and outs of an application domain of interest, possibly before a software engineering effort starts and a production tool is developed. However, it is important to keep a clear distinction between prototype coding and the software engineering required to create a robust, reliable tool

that is widely usable, and it is crucial to convey this distinction to users. Prototype coding is typically more explorative in nature and might lack normal reliability measures, such as tests, for maximizing development speed. Conversely, to create reliable software that is usable by others, the requirements for careful planning, specification, testing and verification, are vastly more stringent. The guidelines below address the development of the latter category: building well-engineered, reliable, robust and reusable software with a clearly defined purpose. They are by no mean exhaustive, but aim at rather highlighting factors that will be essential to the successful development of scientific software for big data.

It is worth noting that practical realities of research software development, such as limited funding and publication pressure, often favors the prototype modality. Simply put, research funding and scientific reward systems have historically discouraged the creation of production-quality software as being non-essential; if a set of code is able to perform the specific task necessary, under the specific environmental context, then it is good enough for inclusion within a publication and it is best to publish as quickly as possible. Given these systematic conditions, it is understandable that software quality and usability are often not pursued, however it is a hindrance to scientific progress and, while we should demand better, at least the code is available. Furthermore, there is often the case that software begins as a prototype, but then becomes production-oriented through a process of continuous improvement. These agile approaches can be powerful and effective processes for creating robust software that meet the evolving needs of a user base. This manuscript is not intended to discourage the creation of prototypes nor the use of agile software development practices, as the authors continue to utilize these approaches when advantageous to a project. However, these guidelines are helpful to keep in mind during initial prototyping, and it should be converged upon in cases when a prototype becomes production-quality distributed software.

Guidelines

Guideline 1: Failing to Plan is Planning to Fail

Spend adequate time designing the scope and expectations of your project. A successful scientist does not simply run to the lab bench with a vague idea of an outcome and start mixing colorful liquids together with the hope of producing something useful. This same type of careful and mindful planning must be engaged in before developing computational tools. Before a single line of code is written, spec out exactly what your tool needs to do. Define all use cases, input datasets and datatypes, configurable parameters, set of actions, and desired outputs and types. Anticipate what is beyond the current scope of your software, what use cases, data types, configurations, sound interesting but fall beyond the initial scope of your software. Sketching out the components and dataflows of the software on a whiteboard, or on paper, can be particularly helpful at this stage. Clearly define the scope of your work, and anticipate corner cases that you think you might need to account for; e.g. are you interested in a circular genome that is going to be represented as a linear sequence for input? Finally, speculate on the factors that will indicate that the software needs to be refactored, replaced or retired.

Once all of the specifications are set, it is time to search, and search thoroughly, to see if there is an open source tool with clear measures of reliability, such as automated software tests, that does what you want, or something very similar. A good place to start is simply to google your initial question or navigate StackOverflow - it is very likely that someone already bumped into the same or a similar problem. If a reliable tool exists that does what you want, consider using it, even if it requires using several tools pipelined together. If there is a reliable tool that exists

that does something close to what you need, consider modifying it. There is no need to design a brand-new blue bicycle, if you can paint an existing white one and achieve the same goal. When assessing the quality of an existing tool, you should follow the same guidelines that are laid out here for designing your own software: 1. Can you understand what the code does, so that you can verify its logic and assumptions, can fix bugs, and improve the code if required? 2. Does the software contain tests? 3. Does it have a high test coverage? 4. Does it use continuous integration? 5. What other dependencies does it rely on? 6. Are those dependencies deemed robust, reliable software? 7. Are the licensing and implementation compatible with integration in your workflow? For tools that lack the required reliability measures such as software tests, consider contributing these missing tests yourself, thereby helping both yourself and other potential users of that software. When doing this mining work, do not hesitate to contact the developers of other tools or users facing the same need as you. Interacting with the community will help better define the project.

The reason for doing such careful and exhaustive research of existing software is that the cost of maintaining yet another software is almost always vastly underestimated. Therefore, generally only consider developing new software if there are clear signs that you can lessen the maintenance burden by creating a drastically improved or simplified implementation that makes it easier to understand, test, and refactor the code, and that such improvements are not possible with the existing software itself. Although outside of the scope of this manuscript, there are several significant and valid alternative reasons for reimplementing code, such as learning exercises, to explore and validate expected or presumed algorithmic behavior, and so forth. Finally, should you decide to develop a new tool, there might be important lessons to be learned from, or parts of the code that can be reused from, existing open source tools. Once you have exhausted all existing options, it is time to move on to the next step.

Guideline 2: Build Test-driven trustworthy code

Instead of coding and then testing, approach from the other way around: before you can begin to convince others that your software implementation is correct, you need to prove it to yourself. Obtain or create a set of input files, and design a set of outputs that are expected to be created across various parameter settings. By relying on sets of known inputs and outputs designed from the planned requirements, you can be confident that the results generated by your software will match the intended requirements. Additionally, test-driven development forces coding efforts to concentrate on features that are needed according to the well-planned requirements, preventing feature creep. Looking at the metaphorical answer at the back of the book helps steering development, thereby creating more precise code.

The "tests first" mantra should drive the development of every aspect from coding to deployment. From the very beginning to the very end, unit tests should be designed to test individual methods and classes while integration and functional testing should be used to confirm the overall correctness of the final application. A gold standard for test suites is to start with a clean environment, install your software, execute your code, and then verify correctness. Testing may also include syntax checking, test coverage checking, checking against coding conventions (often called "linting"), and automatic memory leak detection. It is also good practice to provide negative tests, which will ensure that errors are properly handled. There are many tools that can help testing for virtually every programming language. Take advantage of existing test harnesses and continuous integration suites, such as Jenkins [17], Travis [18], and CircleCI [19].

Guideline 3: Denominate Mindfully and Code for the Future

Naming things is hard. When writing software, there are many things that will need to be named, such as functions, variables, classes, parameters, program arguments, and so on. Names must be self-explanatory to the ones that will be interacting with them. They should describe the intended purpose and not implementation details. While computers don't care, a method named '`do_thing()`' is not descriptive to an individual that may need to read, maintain, or modify the code later.

Names should be chosen very carefully - mind for typos - so that they will not need to be changed in the future, which could result in backwards incompatibility. You should avoid renaming user-exposed items as much as possible and must never reuse or repurpose existing or previously used short or long argument names. For example, if an argument was accessible using '`-o / --orthogonalOption`', you cannot decide later to use '-o' to specify the output filename.

While your code may seem perfectly clear at time of writing, things will be very different in five to ten years - or simply a few days before an important deadline where everything breaks. Give space to your code, organize it so that it is compartmented, clear, and pleasant to read. Making your code understandable to all will help others finding bugs and implementing features. It will also facilitate collaborative development and take some maintenance work off your shoulders.

Guideline 4: Stick to Standards

Stick to standards: standard file types, standard ontologies (e.g., EDAM [20]), standard command line arguments and naming schemes (`--help / -h, --version / -v`), and do not create your own dialect or derivative file format. By utilizing standard file formats, a software tool is made inherently interoperable, allowing simplified use within existing analysis workflows. Adopting standard ontologies allows tools to be easily discoverable. At a minimum, you should provide `--version` and `--help` commands in every tool. The help should provide enough details for a typical user to be able to make use of the tool. Include a `--version` command to enable reproducibility through capturing the version number of your tool easily. Try to avoid defining the version number at multiple locations within your code. Instead, design your code in such a way that the version definition is declared once and then referenced as needed. If possible, try to include the version control system revision number into the output of the version (sometool v1.2.3-5483e9f5bf4d725e3). Take advantage of semantic versioning [21] similar to MAJOR.MINOR.PATCH, where changes in PATCH indicate backwards compatible bug fixes, MINOR indicates backwards compatible feature additions, and MAJOR can indicate large changes that may not be backwards compatible.

Of particular importance is to take advantage of language specific coding standards, as any deviation will make future maintenance much more difficult. For example, in Python, this can mean using indentation of four spaces, not three, not five, not tabs, and certainly not mixed. In Perl, this can mean using parentheses for readability even when not strictly required. When using scripting languages, the shebang is a powerful single line of code, but you should use it in a correct way. Use `` `/usr/bin/env python` `` or `` `/usr/bin/env perl` `` instead of `` `/usr/bin/perl` ``. It is not guaranteed that Perl is installed in `` `/usr/bin/` ``, especially when

using specific versions of a software in virtual environments, such as those created by Conda [22]. In all cases, make proper use of exit codes to report the terminal status of your tool; a 0 means everything in the world is good, and anything other than a 0 is an error.

Make use of previously existing stable libraries and packages (e.g. Biopython [23], pysam [24], BioPerl [25], SeqAn [26], BioJava [27], BioJS [28]) whenever possible including the specific version of each required dependency in the installation methodology. Beware of licensing conflicts. For libraries and packages that are not available in stable repositories or standard distribution channels such as those mentioned above, but e.g. only in a single developer's git repository, it is recommended to ensure the availability of the library or package in some way, such as by submitting the package to a stable repository or distribution channel, making a fork of the library into a repository that you as developer have control over, or additional backup methodology. Be sure to include an accepted standard open-source LICENSE with your code. Adopting a customized or oddball license can lead to issues downstream and greatly hinder community acceptance. The Open Source Initiative (OSI), a non-profit organization that promotes and protects open source software, projects and communities, provides guidance on selecting from a list of approved open source licenses (https://opensource.org/licenses).

Adopt well-established standard frameworks and approaches to handle common problems. For example, if you have an easily parallelizable problem, e.g. if your tool consumes a multiple FASTA file and operates on each unit independently, it is a good idea to split the FASTA file into small chunks to speed up your tool by using multiple processing cores. However, do not reinvent the wheel when adding these optimizations, instead make use of existing reliable tools and frameworks; in particular, become well-versed in common GNU/Linux utilities such as `awk`, `grep`, `split`, `parallel`, etc. to handle large files.

Guideline 5: Choose Sane Defaults, but Allow Overriding

When providing default values for parameters, choose settings that make the most sense for the typical application of the tool. In the help for your tool provide the default value used, and the impacts that changing it will have. Allow default values to be overridden by environmental variables and by supplying a command-line argument, in increasing order of preference.

For example, when creating temporary intermediate files, use predefined libraries from your favorite programming language (like `tempfile` in python), which will place files in the location defined using operating system defined mechanisms. This enables the cluster admin to have the option to assert the use of fast local hard-drives for temporary storage, which can drastically speed up IO heavy computational tasks. While the temporary directory is often controllable by using an environment variable (`$TMPDIR`), it can also be useful to allow this to be changed using a tool specific environment setting (e.g. *`$MY_TOOL_TEMP_DIR`*) and by passing an explicit argument to the tool (e.g. `--tmpdir`). This gives the system administrator, users, and workflow management systems multiple injection points resulting in the greatest flexibility to control data flow. When there are multiple ways to specify a parameter value, log the final value utilized to the standard application log when the tool starts, so that it is easy to spot any values being overridden by accident. At completion, log all values and assumptions used for traceability and reproducibility of the results.

Guideline 6: Make no Assumptions

“Never ASSUME, because when you ASSUME, you…” Assumptions can result in cryptic errors, or worse, erroneous results that are reported without notification. Don’t assume that the user will have a home directory for storing files and configurations (e.g. `.programNameConfig`). In many cluster environments, jobs and tasks may not have access to a home directory. And they almost certainly do not have *your* home directory, so please do not hardcode that. As a general rule, you will be surprised by how creative your users will be at using your code and breaking it. If something can go wrong, it will - Occam's razor programming will save you countless hours of debugging.

Do not rely solely on file extensions to determine the filetype. As a default setting this is good, but offer alternative ways to instruct your program which file type is provided. File extensions are often not standardized. Moreover, data management systems might store the filetype and other metadata in a database separate from the dataset contents, without a file extension or human-readable filename. Often these datasets are identified by a hashsum or a UUID. Enabling compatibility with object-based storage is a worthwhile goal. Similarly, if you define a novel file format, take effort to enable automatic type detection systems the ability to identify files, such as by making use of magic numbers. Data management systems might also need to use a modified filename while a file is being created, to enable the separation of finished and unfinished files, such as in the case of a crashed workflow run, so that unfinished files are not erroneously reused when the workflow is restarted.

Ideally, allow the ability to customize completely the filename of every input file and associated metadata file, and every output file and associated metadata file generated by the tool. For

example, it is bad practice to only allow the tool to accept a file named `input.bam` that is located in the current working directory. If for any reason, the tool cannot reasonably take the exact file name for every output file (e.g. when the number of outputs is unknown, or very large) allow the user to specify both an output directory and a file name pattern.

Guideline 7: Be One with the Streams

You can see every tool as a filter: it turns an input into an output. Whenever possible, make your tool streaming aware. Filesystem IO is increasingly becoming a bottleneck in modern computing. Allow the primary input and output datasets to be consumed and produced as streams, which are only temporarily stored in random-access memory (RAM), so that they can be used effectively with redirection, and, more importantly, pipes (|), preventing the need to read and write from disk when the tool is used as part of a multi-tool process. When working with streams, be aware that for input and output, text is often king. Furthermore, the use of standard stream-aware Linux utilities, which primarily operate in the text-based realm, can be mightily powerful for processing and filtering.

Guideline 8: Metadata is Valuable

Metadata is as important as the primary dataset. Try to stick to existing standard file formats, but if it is necessary to create a new or extend an existing file format, be sure to make

provisions for storing metadata. Include enough information to unambiguously identify the filetype, version, and meaning behind values (e.g. column names). It is a good practice to also include information about the generating program, including version and complete set of parameters. Do what you can to keep data and its associated metadata as closely attached as possible, and in sync. If at all possible, avoid using metadata files (`file.ext` and `file.ext.ext2`), but include metadata within a metadata section or header of the dataset. This ensures that data and metadata remain in sync and do not accidentally diverge, e.g. by accidentally moving a subset of the files around on a file system. If separate metadata files need to be created, it can in some cases be helpful to package data and metadata together in an archive file or container format such as `tar`, `zip`, or `HDF5`.

Guideline 9: Users come first

Computational tools must be usable by the widest range of users. The tool should be executable on user provided data without requiring them to jump through numerous steps that could be easily automated. One common example of this is requiring input datasets and metadata to use a particular filename or follow a specific pattern. The user should be able to provide file names directly and be able to make use of standard command-line interpreter features and shellisms such as wildcards and tab-completion. The use of a file to specify input parameters, instead of accepting command line arguments is also discouraged, as it unnecessarily complicates the notion of input files by creating two different classes of input files. Making it easy to run your tools will be rewarded by increased adoption by the community and decreased support and maintenance burden.

Installation of the computational tool is likewise important, installation procedures for a tool and or its dependencies can become a challenge for users of different backgrounds [29]. Simply put, if a tool cannot be installed, it cannot be used. Try to make use of the default installation mechanism of the programming language. There are many tools that do not obey these rules and require patching and additional scripting in order to be installed. Do not include compiled binaries nor external source code within your version control system. Binaries should be either generated from the code base or should be provided by a package management system. External source code should be resolved with the installation mechanism (e.g. GNU Autotools [30], pip [31], Pom/Maven [32], etc.). The use of Conda, particularly conda-forge [33] and Bioconda [34], has been shown to be very effective in providing versioned ready-to-go cross-platform tool environments. Finally, mind the system requirements of servers handling sensitive data: they generally offer limited connection to the internet and do not allow running all kinds of containers.

Guideline 10: Documentation is Paramount

Providing good end-user documentation is essential to enable users to effectively use any computation tool. Simplified, succinct documentation should be provided onscreen with a `--help` argument to the tool. More in depth documentation should be provided via man pages, web pages, and PDF documents. Providing usage tutorials, or vignettes, that walk through typical use cases is particularly beneficial to users. A good approach is to embed the documentation within a “doc” directory of the source code in a human-readable language such

as markdown. These markdown files can then be automatically converted into stylized HTML or PDF documents. It is a good practice to provide clear support avenues such as a mailing list, forum, and issue tracker where users can request assistance and report bugs. These services are greatly simplified by open-source public code repositories, such as GitHub [35], GitLab [36], Bitbucket [37], etc. Finally, consider including a CONTRIBUTING document that informs other community developers how they can commit code or otherwise help with your project.

Take advantage of the version control system to transparently document every change in the code. The version control system is as important when developing software as a lab book during an experiment. Provide an accurate, but concise changelog between versions. This changelog should be easily understandable by the end user, and highlight changes of importance, including changes in behavior and default settings, deprecated and new parameters, etc. It should not include changes that, while important, do not impact the end user, such as internal architectural changes. In other words, this is not simply the unannotated output of `git log`.

Conclusions

Progressing from writing simple one-off scripts to developing truly useful and reusable computational tools requires substantially more planning and increased considerations. Do not rush through the initial steps. Time spent designing the scope and expectations of a project, including input datasets and formats, configurable parameters, and desired outputs and output types is time well-spent. By starting with a set of tests, you can ensure that your tool is

functionally correct. Adopt and use existing, well-established, tools, file formats, and other standards whenever they are available. Do not make changes that will break backwards compatibility, except when absolutely necessary, and increment the software versions appropriately. Be sure to choose sane default values for tool parameters, but allow users to easily change their values. Do not make any assumptions about the local compute environment or infrastructure. Making your software stream aware goes a long way to encouraging its use on HPC resources and incorporation into existing computational pipelines. Metadata is as important as the primary datasets, be sure to treat it with the same care, and try to keep it as closely connected to the primary data as possible. Always be aware of the various types of users that your software will have, and tailor your tool, development approaches, and documentation to support them. As you continue to advance from a simple script writer to a software developer, do not unnecessarily fret about unknown bugs or deficiencies in coding ability, knowledge, or style, as everyone starts somewhere, but be sure to avoid developing a bad code-ego [38], keep learning about open source best practices (e.g. https://opensource.guide/, http://osodos.org), and do not hesitate to reach out to the community.

Declarations

Ethics approval and consent to participate

Not applicable.

Consent for publication

Not applicable.

Availability of data and material

Not applicable.

Competing interests

The authors declare that they have no competing interests.

Funding

Supported with funds provided by the Cleveland Clinic, and the European Union’s Horizon 2020 research and innovation programme under grant agreement No 654241 for the PhenoMeNal project.

Authors' contributions

All authors wrote the manuscript. All authors read and approved the final manuscript.

Acknowledgements

Not applicable.

References

1. Piccolo SR, Frampton MB. Tools and techniques for computational reproducibility. Gigascience [Internet]. 2016;5:30. Available from: http://dx.doi.org/10.1186/s13742-016-0135-4

2. Sandve GK, Nekrutenko A, Taylor J, Hovig E. Ten simple rules for reproducible

computational research. PLoS Comput Biol [Internet]. 2013;9:e1003285. Available from: http://dx.doi.org/10.1371/journal.pcbi.1003285

3. Nekrutenko A, Team, Galaxy, Goecks J, Taylor J, Blankenberg D. Biology needs evolutionary software tools: Let's build them right. Mol Biol Evol [Internet]. 2018 [cited 2018 Apr 25]; Available from: https://academic.oup.com/mbe/advance-article/doi/10.1093/molbev/msy084/4983859

4. Jin X, Khatwani C, Niu N, Wagner M, Savolainen J. Pragmatic Software Reuse in Bioinformatics: How Can Social Network Information Help? Software Reuse: Bridging with Social-Awareness [Internet]. Springer International Publishing; 2016. p. 247–64. Available from: http://dx.doi.org/10.1007/978-3-319-35122-3_17

5. Perez-Riverol Y, Gatto L, Wang R, Sachsenberg T, Uszkoreit J, Leprevost F da V, et al. Ten Simple Rules for Taking Advantage of Git and GitHub. PLoS Comput Biol [Internet]. 2016;12:e1004947. Available from: http://dx.doi.org/10.1371/journal.pcbi.1004947

6. Prlić A, Procter JB. Ten simple rules for the open development of scientific software. PLoS Comput Biol [Internet]. 2012;8:e1002802. Available from: http://dx.doi.org/10.1371/journal.pcbi.1002802

7. Wilson G, Aruliah DA, Brown CT, Chue Hong NP, Davis M, Guy RT, et al. Best practices for scientific computing. PLoS Biol [Internet]. 2014;12:e1001745. Available from: http://dx.doi.org/10.1371/journal.pbio.1001745

8. Taschuk M, Wilson G. Ten simple rules for making research software more robust. PLoS Comput Biol [Internet]. 2017;13:e1005412. Available from: http://dx.doi.org/10.1371/journal.pcbi.1005412

9. Lawlor B, Walsh P. Engineering bioinformatics: building reliability, performance and productivity into bioinformatics software. Bioengineered [Internet]. 2015;6:193–203. Available from: http://dx.doi.org/10.1080/21655979.2015.1050162

10. Afgan E, Baker D, Batut B, van den Beek M, Bouvier D, Cech M, et al. The Galaxy platform for accessible, reproducible and collaborative biomedical analyses: 2018 update. Nucleic Acids Res [Internet]. 2018; Available from: http://dx.doi.org/10.1093/nar/gky379

11. Wolstencroft K, Haines R, Fellows D, Williams A, Withers D, Owen S, et al. The Taverna workflow suite: designing and executing workflows of Web Services on the desktop, web or in the cloud. Nucleic Acids Res [Internet]. 2013;41:W557–61. Available from: http://dx.doi.org/10.1093/nar/gkt328

12. Reich M, Liefeld T, Gould J, Lerner J, Tamayo P, Mesirov JP. GenePattern 2.0. Nat Genet [Internet]. 2006;38:500–1. Available from: http://dx.doi.org/10.1038/ng0506-500

13. Di Tommaso P, Chatzou M, Floden EW, Barja PP, Palumbo E, Notredame C. Nextflow enables reproducible computational workflows. Nat Biotechnol [Internet]. 2017;35:316–9. Available from: http://dx.doi.org/10.1038/nbt.3820

14. Köster J, Rahmann S. Snakemake--a scalable bioinformatics workflow engine. Bioinformatics [Internet]. 2012;28:2520–2. Available from: http://dx.doi.org/10.1093/bioinformatics/bts480

15. Sadedin SP, Pope B, Oshlack A. Bpipe: a tool for running and managing bioinformatics pipelines. Bioinformatics [Internet]. 2012;28:1525–6. Available from: http://dx.doi.org/10.1093/bioinformatics/bts167

16. Brandt J, Bux M, Leser U. Cuneiform: a Functional Language for Large Scale Scientific Data Analysis. EDBT/ICDT Workshops [Internet]. 2015. p. 7–16. Available from: http://ceur-ws.org/Vol-1330/paper-03.pdf

17. Jenkins [Internet]. Jenkins. [cited 2018 Jun 20]. Available from: https://jenkins.io/

18. Travis CI - Test and Deploy Your Code with Confidence [Internet]. [cited 2018 Jun 20]. Available from: https://travis-ci.org/

19. Continuous Integration and Delivery [Internet]. CircleCI. [cited 2018 Jun 20]. Available from: https://circleci.com/

20. Ison J, Kalas M, Jonassen I, Bolser D, Uludag M, McWilliam H, et al. EDAM: an ontology of bioinformatics operations, types of data and identifiers, topics and formats. Bioinformatics [Internet]. 2013;29:1325–32. Available from: http://dx.doi.org/10.1093/bioinformatics/btt113

21. Preston-Werner T. Semantic versioning, 2015. URL http://semver org/ Visited on [Internet]. 2015;12–4. Available from: https://semver.org/

22. — Conda [Internet]. [cited 2018 Jun 20]. Available from: https://conda.io/

23. Cock PJA, Antao T, Chang JT, Chapman BA, Cox CJ, Dalke A, et al. Biopython: freely available Python tools for computational molecular biology and bioinformatics. Bioinformatics [Internet]. 2009;25:1422–3. Available from: http://dx.doi.org/10.1093/bioinformatics/btp163

24. pysam [Internet]. Github; [cited 2018 Jun 20]. Available from: https://github.com/pysam-developers/pysam

25. Stajich JE, Block D, Boulez K, Brenner SE, Chervitz SA, Dagdigian C, et al. The Bioperl toolkit: Perl modules for the life sciences. Genome Res [Internet]. 2002;12:1611–8. Available from: http://dx.doi.org/10.1101/gr.361602

26. Reinert K, Dadi TH, Ehrhardt M, Hauswedell H, Mehringer S, Rahn R, et al. The SeqAn C++ template library for efficient sequence analysis: A resource for programmers. J Biotechnol [Internet]. 2017;261:157–68. Available from: http://dx.doi.org/10.1016/j.jbiotec.2017.07.017

27. Holland RCG, Down TA, Pocock M, Prlić A, Huen D, James K, et al. BioJava: an open-source framework for bioinformatics. Bioinformatics [Internet]. 2008;24:2096–7. Available from: http://dx.doi.org/10.1093/bioinformatics/btn397

28. Yachdav G, Goldberg T, Wilzbach S, Dao D, Shih I, Choudhary S, et al. Anatomy of BioJS, an open source community for the life sciences. Elife [Internet]. 2015;4. Available from: http://dx.doi.org/10.7554/eLife.07009

29. Gruening B, Sallou O, Moreno P, da Veiga Leprevost F, Ménager H, Søndergaard D, et al. Recommendations for the packaging and containerizing of bioinformatics software. F1000Res [Internet]. 2018 [cited 2018 Jun 20];7. Available from: https://f1000research.com/articles/7-742/v1/pdf

30. Autotools FAQ [Internet]. [cited 2018 Jun 20]. Available from: https://www.gnu.org/software/automake/faq/autotools-faq.html

31. pip [Internet]. PyPI. [cited 2018 Jun 20]. Available from: https://pypi.org/project/pip/

32. Porter B, van Zyl J, Lamy O. Maven – Welcome to Apache Maven [Internet]. [cited 2018 Jun 20]. Available from: https://maven.apache.org/

33. conda-forge | community driven packaging for conda [Internet]. [cited 2018 Jun 20]. Available from: https://conda-forge.org/

34. Grüning B, Dale R, Sjödin A, Chapman BA, Rowe J, Tomkins-Tinch CH, et al. Bioconda: sustainable and comprehensive software distribution for the life sciences. Nat Methods [Internet]. 2018;15:475–6. Available from: http://dx.doi.org/10.1038/s41592-018-0046-7

35. Build software better, together [Internet]. Github; [cited 2018 Jun 20]. Available from: https://github.com

36. The only single product for the complete DevOps lifecycle - GitLab [Internet]. GitLab. [cited 2018 Jun 20]. Available from: https://about.gitlab.com/

37. Atlassian. Bitbucket | The Git solution for professional teams [Internet]. Bitbucket. [cited 2018 Jun 20]. Available from: https://bitbucket.org/

38. Weinberg GM. The psychology of computer programming [Internet]. Van Nostrand Reinhold; 1971. Available from: https://openlibrary.org/books/OL7528135M/Psychology_of_Computer_Programming

 Click here to access/download;Personal Cover;coding_bd_response_to_reviewers.pdf

Dear Scott,

We want to take this opportunity to thank you and the referees for reviewing this manuscript. We are greatly appreciative of the time and effort expended and feel that the provided comments, suggestions, and queries have resulted in an improved submission.

We have replied to the reviewers' comments inline below, and have made referenced changes in the submitted revision.

Please do not hesitate to contact me if there are additional comments or concerns.

Sincerely,

Daniel Blankenberg
blanked2@ccf.org
Office: 216-445-4336
Cell: 216-212-2211

Reviewer #1: This is a nice, neat article about best practices in software engineering as applied to the development of command-line utilities for scientific data processing. In general, there is little with which to disagree in the article. However, I would recommend the authors consider a few changes to clarify the central message. Firstly, although the title says "Software engineering for scientific big-data analysis", there was little in the guidelines presented that seemed to me to be specific to big-data analysis. The authors might consider highlighting which aspects of the software engineering guidelines they present are uniquely relevant to big data.

This is a good point. In fact, we would agree that the guidelines presented here are applicable and helpful for software engineering in general. However, the nature of big data analysis with increased concerns such as dataset and workflow/pipeline complexity, availability and costs of computational resources, etc., necessitates increased vigilance. The cost of having inadequate or erroneous software in big data analysis is amplified while, in many cases, becoming simultaneously more difficult to uncover. It is our goal to raise awareness and address these concerns under this context.

*Secondly, the authors distinguish at an early stage between software prototyping vs. production. However, in research contexts it is particularly commonly the case that the prototype *becomes* the production software in an evolutionary fashion, without sufficient time or funding to pause to consider how to engineer a production version. Of course, this is not because individual researchers necessarily lack the expertise to develop production-level software. Rather, it reflects the broader, environmental system of research funding and reward schemas, which are often outside of the control of individual researchers. I wonder if it would be useful to discuss this issue, given the central focus of your article on the processes involved in careful production-level processes.*

We have added an additional paragraph to the background section discussing the usefulness of prototypes, the common case when a prototype converges into a distributed software package, and the realities of creating production-quality software under current funding and research-effort reward paradigms.

Reviewer #2: In this paper, Gruning et al provide their thoughts on "Software engineering
for scientific big-data analysis." It covers a variety of strategies to produce reliable software for scientific data analysis at scale.

This was a tough paper to review. About half the time I found myself cheering, about half the time I disagreed with some details, and about half the time I found myself wondering why the authors were making this point. (Yes, that adds up to 1.5 of the time.)

I'll start with my major disagreements --

There seems to be no allowance int his paper for the view that you can produce robust software while coding your way to a better understanding of the problem. I would term this an "agile" approach, and it's something my group does regularly; it seems to work pretty well and reaches the same end point these authors are suggesting, of reliable and robust software that can be operated at scale. It just does so circuitously. I don't even think the authors would disagree with it. But it is simply not discussed in this paper.

We have added an additional paragraph to the background section discussing the usefulness of prototypes, the common case when a prototype converges into a distributed software package, and the realities of creating production-quality software under current funding and research-effort reward paradigms.

I think in general that this paper veers wildly between high level statements (robust software is important! create reliable software! plan ahead!) and minutia (use THESE command line options). Fine, but we're missing the middle view. I'm really not sure how to fix this, so I'll just leave it as an opinion for them to respond to!

This is a valid concern. The intention of this manuscript is to provide an easily consumable list of guidelines, along with illustrative examples. This is a hard balance to strike, especially when considering the open-endedness of potential programming languages, a variety of software development paradigms, text length and conciseness, etc. If there are specific items of particular concern, we would be happy to address them.

*The authors laud reuse (but I'm curious how often they've been able to reuse existing software and adapt it - we virtually never can?) and ignore the *significant* value of reimplementing solutions from*

scratch, where you can detect implementation oddities that went undescribed in the initial paper. These tend not to be published directly, but you can look at (e.g.) the Oyster River Protocol meta-analysis paper by MacManes to see how undescribed algorithmic and parameter choices can have profound downstream impacts on outcomes. The other downside of reuse that is not really mentioned is the substantial complexity of so-called "general purpose" libraries; we don't use BioPython in my lab because we don't need 99% of it, and don't want to have to debug it.

These are excellent points. We completely agree that there are often times when reuse is not the best course of action, and in guideline 1 we present details on what to look for when judging whether or not to reuse a specific set of software.

These requirements set a relatively high bar for reuse. So, while we laud reuse and assert that it should be the default first approach, there are many occasions where it may not be prudent. For example, in the case of a many-domain general purpose library, it may not pass several of these requirements, including test coverage, understandability of code and ease of debugging, etc.

Addressing the specific case of the impacts of undescribed algorithmic and parameter choices of existing code can also be addressed by test coverage (having the correct set of tests based upon intended outcome), code clarity (being able to see implementation oddities), etc.

However, there are important reasons to reimplement code and we do not mean to imply that there is no value in it. We have added the following text to convey this sentiment: “Although outside of the scope of this manuscript, there are several significant and valid alternative reasons for reimplementing code, such as learning exercises, to explore and validate expected or presumed algorithmic behavior, and so forth.”

In some sense, a lot of this is covered in Morgan Taschuk and Greg Wilson's piece on making research software robust. Maybe the authors could focus on what they DON'T cover?

Morgan Taschuk and Greg Wilson provide a valuable resource with their paper, and we very early-on recommend its reading. Unfortunately, some overlap is unavoidable in order for this manuscript to stand on its own and to best service the reader; or, perhaps, it is fortunate, as the overlaps demonstrates importance and, potentially, aids with retention and adoption through multiple exposure. We view this manuscript as complementary to previous efforts, with each contributing individually, in addition to a common core.

Minor issues --

there's no guidance on choosing a license, at all. At the very least some citations would probably be a good idea. And please mention OSI licensing; there are a list of approved open source licenses to use, period.

We completely agree that the importance of carefully selecting a license is far too often undervalued or misunderstood. We added additional information about open source licensing to guideline 4 (new in bold): “Be sure to include an accepted standard open-source LICENSE with your code. Adopting a customized or oddball license can lead to issues downstream and greatly hinder community acceptance. **The Open Source Initiative (OSI), a non-profit organization that promotes and protects open source software, projects and communities, provides guidance on selecting from a list of approved open source licenses (https://opensource.org/licenses).**”

The statement "use containers" is somewhat undermined by "sometimes you can't".

Yes. This is true in the case of containers, and also true for the case of many other concerns in software. The intricacies involved, and a myriad of possible design choices, in building software make declaring steadfast rules difficult, which is why we present our recommendations as guidelines.
