# Supplementary material for: Software engineering for scientific big data analysis
Source: Gigascience. 2019 May 23;8(5):giz054. doi: 10.1093/gigascience/giz054 (PMC6532757; doi:10.1093/gigascience/giz054)
Supplement: giz054_Response_to_Reviewer_Comments_Original_Submission [file giz054_response_to_reviewer_comments_original_submission.pdf]

Author's Response To Reviewer Comments

Close

PDF also attached.

Dear Scott,

We want to take this opportunity to thank you and the referees for reviewing this manuscript. We are greatly appreciative of the time and effort expended and feel that the provided comments, suggestions, and queries have resulted in an improved submission.

We have replied to the reviewers' comments inline below, and have made referenced changes in the submitted revision.

Please do not hesitate to contact me if there are additional comments or concerns.

Sincerely,

Daniel Blankenberg
blanked2@ccf.org
Office: 216-445-4336
Cell: 216-212-2211

Reviewer #1: This is a nice, neat article about best practices in software engineering as applied to the development of command-line utilities for scientific data processing. In general, there is little with which to disagree in the article. However, I would recommend the authors consider a few changes to clarify the central message. Firstly, although the title says "Software engineering for scientific big-data analysis", there was little in the guidelines presented that seemed to me to be specific to big-data analysis. The authors might consider highlighting which aspects of the software engineering guidelines they present are uniquely relevant to big data.

This is a good point. In fact, we would agree that the guidelines presented here are applicable and helpful for software engineering in general. However, the nature of big data analysis with increased concerns such as dataset and workflow/pipeline complexity, availability and costs of computational resources, etc., necessitates increased vigilance. The cost of having inadequate or erroneous software in big data analysis is amplified while, in many cases, becoming simultaneously more difficult to uncover. It is our goal to raise awareness and address these concerns under this context.

Secondly, the authors distinguish at an early stage between software prototyping vs. production. However, in research contexts it is particularly commonly the case that the prototype *becomes* the production software in an evolutionary fashion, without sufficient time or funding to pause to consider how to engineer a production version. Of course, this is not because individual researchers necessarily lack the expertise to develop production-level software. Rather, it reflects the broader, environmental system of research funding and reward schemas, which are often outside of the control of individual researchers. I wonder if it would be useful to discuss this issue, given the central focus of your article on the processes involved in careful production-level processes.

We have added an additional paragraph to the background section discussing the usefulness of prototypes, the common case when a prototype converges into a distributed software package, and the realities of creating production-quality software under current funding and research-effort reward paradigms.

Reviewer #2: In this paper, Gruning et al provide their thoughts on "Software engineering for scientific big-data analysis." It covers a variety of strategies to produce reliable software for scientific data analysis at scale.

This was a tough paper to review. About half the time I found myself cheering, about half the time I disagreed with some details, and about half the time I found myself wondering why the authors were making this point. (Yes, that adds up to 1.5 of the time.)

I'll start with my major disagreements --

There seems to be no allowance int his paper for the view that you can produce robust software while coding your way to a better understanding of the problem. I would term this an "agile" approach, and it's something my group does regularly; it seems to work pretty well and reaches the same end point these authors are suggesting, of reliable and robust software that can be operated at scale. It just does so circuitously. I don't even think the authors would disagree with it. But it is simply not discussed in this paper.

We have added an additional paragraph to the background section discussing the usefulness of prototypes, the common case when a prototype converges into a distributed software package, and the realities of creating production-quality software under current funding and research-effort reward paradigms.

I think in general that this paper veers wildly between high level statements (robust software is important! create reliable software! plan ahead!) and minutia (use THESE command line options). Fine, but we're missing the middle view. I'm really not sure how to fix this, so I'll just leave it as an opinion for them to respond to!

This is a valid concern. The intention of this manuscript is to provide an easily consumable list of guidelines, along with illustrative examples. This is a hard balance to strike, especially when considering the open-endedness of potential programming languages, a variety of software development paradigms, text length and conciseness, etc. If there are specific items of particular concern, we would be happy to address them.

The authors laud reuse (but I'm curious how often they've been able to reuse existing software and adapt it - we virtually never can?) and ignore the *significant* value of reimplementing solutions from scratch, where you can detect implementation oddities that went undescribed in the initial paper. These tend not to be published directly, but you can look at (e.g.) the Oyster River Protocol meta-analysis paper by MacManes to see how undescribed algorithmic and parameter choices can have profound downstream impacts on outcomes. The other downside of reuse that is not really mentioned is the substantial complexity of so-called "general purpose" libraries; we don't use BioPython in my lab because we don't need 99% of it, and don't want to have to debug it.

These are excellent points. We completely agree that there are often times when reuse is not the best course of action, and in guideline 1 we present details on what to look for when judging whether or not to reuse a specific set of software.

These requirements set a relatively high bar for reuse. So, while we laud reuse and assert that it should be the default first approach, there are many occasions where it may not be prudent. For example, in

the case of a many-domain general purpose library, it may not pass several of these requirements, including test coverage, understandability of code and ease of debugging, etc.

Addressing the specific case of the impacts of undescribed algorithmic and parameter choices of existing code can also be addressed by test coverage (having the correct set of tests based upon intended outcome), code clarity (being able to see implementation oddities), etc.

However, there are important reasons to reimplement code and we do not mean to imply that there is no value in it. We have added the following text to convey this sentiment: “Although outside of the scope of this manuscript, there are several significant and valid alternative reasons for reimplementing code, such as learning exercises, to explore and validate expected or presumed algorithmic behavior, and so forth.”

In some sense, a lot of this is covered in Morgan Taschuk and Greg
Wilson's piece on making research software robust. Maybe the authors
could focus on what they DON'T cover?

Morgan Taschuk and Greg Wilson provide a valuable resource with their paper, and we very early-on recommend its reading. Unfortunately, some overlap is unavoidable in order for this manuscript to stand on its own and to best service the reader; or, perhaps, it is fortunate, as the overlaps demonstrates importance and, potentially, aids with retention and adoption through multiple exposure. We view this manuscript as complementary to previous efforts, with each contributing individually, in addition to a common core.

Minor issues --

there's no guidance on choosing a license, at all. At the very least some
citations would probably be a good idea. And please mention OSI licensing;
there are a list of approved open source licenses to use, period.

We completely agree that the importance of carefully selecting a license is far too often undervalued or misunderstood. We added additional information about open source licensing to guideline 4 (new in bold): “Be sure to include an accepted standard open-source LICENSE with your code. Adopting a customized or oddball license can lead to issues downstream and greatly hinder community acceptance. The Open Source Initiative (OSI), a non-profit organization that promotes and protects open source software, projects and communities, provides guidance on selecting from a list of approved open source licenses (https://opensource.org/licenses).”

The statement "use containers" is somewhat undermined by "sometimes you can't".

Yes. This is true in the case of containers, and also true for the case of many other concerns in software. The intricacies involved, and a myriad of possible design choices, in building software make declaring steadfast rules difficult, which is why we present our recommendations as guidelines.

Close
