# Supplementary material for: Software engineering for scientific big data analysis
Source: Gigascience. 2019 May 23;8(5):giz054. doi: 10.1093/gigascience/giz054 (PMC6532757; doi:10.1093/gigascience/giz054)
Supplement: giz054_Response_to_Reviewer_Comments_Revision_1 [file giz054_response_to_reviewer_comments_revision_1.pdf]

Author's Response To Reviewer Comments

Close

PDF file containing formatted responses is attached, with a plain text version included below:

Reviewer reports:
Reviewer #2: In the manuscript "Software engineering for scientific big data analysis", the authors lay out a set of rules or guidelines for developing software to aid in, well, big data analysis!

This is revision 1 of the paper.

While I don't entirely agree with the detailed recommendations and could argue strongly about a few of them, that's ok - I think the paper makes a number of valuable recommendations for the field. In particular, the authors have a lot of experience with workflow systems, and their recommendations there mirror my own realizations (which cost me about a decade of software development), so I hope they can get out there!

In the response to reviewers, the authors seem to narrowly address the concern that both reviewers had about the "big design up front" approach suggested in the paper. On reading the revisions, I think the reviewers did a reasonable job of addressing this concern. There are several places where I suggest some modifications or softening of perspective (below), but overall this is a very nice paper.

We are very appreciative of the reviewer’s comments and concerns. We have made each of the suggested modifications, and find them to have enhanced the manuscript and its messaging.

---

p3 line 56, "and it should be converged upon" - remove "it"

Great catch, we have fixed this. Thank you.

p4 line 22-25, suggest modifying this to avoid the requirement of upfront design: "and mindful planning must be engaged in _while_ developing computational tools." then remove "Before a single line of code is written..." The paragraph reads just as well, is less prescriptive, and addresses both reviewers concerns better!

We agree, and have made these changes. Thank you for these tempering suggestions.

p5 line 40, here I would note that the professional software industry is quite bad at determing whether adoption, refactoring, or rewriting is the best approach. It seems odd to argue that scientists should be better than industry here.

We have added a note on the difficulty of making these decisions: “Making this determination is a difficult task, one that can be challenging for even the most seasoned software industry professionals,

and in the end rests on what is essentially a judgement call that should consider all aspects, including not only the code, but also the existing developer and user communities, of the software under consideration.”

p6, line 17: I can think of many situations in which known-good outputs
are virtually impossible to develop, e.g. quite a bit of bioinformatics
research software where approximations and details are unknown until
the analysis is done. I don't want to get too philosophical here, but
I might argue that the point of new Big Data software is to do analysis
jobs that are too big for current software. This leaves the developer
following this recommendation in the odd position of having to develop
known good outputs for simple inputs, which then don't really apply to
the interesting behavior of the program. Good practice, yes (we do this)
but not prescriptive and certainly not complete.

We have added the following text: “Especially in the case of the development of new algorithms and approaches that are only exercised when subjected to real data, it may not be practical to design a full test suite to verify the complete correctness of the approach until after it has been mostly coded and executed. In these cases, it is still important to test as much as possible, as early as possible, with initial tests and then to be sure to establish a test-set that encompasses the entire behavior. ”

(The whole of guideline 2 excludes most existing software in science!
I'm not sure I disagree, but it's a pretty high bar.)

We agree that this is a rather high bar, especially for research-grade software, however, the importance of having accurate and reliable testing cannot be understated. It may be the case that such well-tested software is currently more rare than common, but as a discipline we need to move away from the perception that testing is one of those things we can afford to do last, just ‘to check an item off of a list’, and make these essential controls an integral part of the initial and continuing development process.

---

p7 line 29 - why is breaking backwards compatibility bad? Python does
it regularly (if slowly). The key (in my opinion) is to be aware of it
and deal with it, perhaps through semantic versioning, not to avoid it
at all costs.

I would argue that testing facilitates refactoring, and that's a
real point of guideline 2 and 3 - you want to be able to change your
software without breaking it. Unchanging software is dead.

We absolutely agree. Refactoring and improving software is essential in many cases for it to remain relevant and useful, and, that by utilizing testing facilities, these changes can be performed with relative certainty that the intended functionality remains intact. However, the single ‘variable’ that developers have the least amount of control over is the user. From a typical user’s perspective, the only thing worse than a command-line program that changes its arguments and ‘stops working the way that it used to’, is one that changes its signature and still looks to be working, but is doing something different. Any action that would disrupt or confuse the interaction of the user with the software should be thoroughly considered, perhaps through the lens of a user copy&pasting command-line templates from some (years-old) online tutorial that they stumbled upon.

We have modified the text to be less prescriptive here, while attempting to ensure that users are conspicuously informed of changes.

Close
