# Supplementary material for: Software engineering for scientific big data analysis
Source: Gigascience. 2019 May 23;8(5):giz054. doi: 10.1093/gigascience/giz054 (PMC6532757; doi:10.1093/gigascience/giz054)
Supplement: giz054_Reviewer_2_Report_Original_Submission -- C. Titus Brown — 10/21/2018 Reviewed [file giz054_reviewer_2_report_original_submission.pdf]

Reviewer Report

Title: Software engineering for scientific big data analysis

Version: Original Submission **Date:** 10/21/2018

Reviewer name: C. Titus Brown

Reviewer Comments to Author:

In this paper, Gruning et al provide their thoughts on "Software engineering for scientific big-data analysis." It covers a variety of strategies to produce reliable software for scientific data analysis at scale.

This was a tough paper to review. About half the time I found myself cheering, about half the time I disagreed with some details, and about half the time I found myself wondering why the authors were making this point. (Yes, that adds up to 1.5 of the time.)

I'll start with my major disagreements --

There seems to be no allowance int his paper for the view that you can produce robust software while coding your way to a better understanding of the problem. I would term this an "agile" approach, and it's something my group does regularly; it seems to work pretty well and reaches the same end point these authors are suggesting, of reliable and robust software that can be operated at scale. It just does so circuitously. I don't even think the authors would disagree with it. But it is simply not discussed in this paper.

I think in general that this paper veers wildly between high level statements (robust software is important! create reliable software! plan ahead!) and minutia (use THESE command line options). Fine, but we're missing the middle view. I'm really not sure how to fix this, so I'll just leave it as an opinion for them to respond to!

The authors laud reuse (but I'm curious how often they've been able to reuse existing software and adapt it - we virtually never can?) and ignore the *significant* value of reimplementing solutions from scratch, where you can detect implementation oddities that went undescribed in the initial paper. These tend not to be published directly, but you can look at (e.g.) the Oyster River Protocol meta-analysis paper by MacManes to see how undescribed algorithmic and parameter choices can have profound downstream impacts on outcomes.

The other downside of reuse that is not really mentioned is the substantial complexity of so-called "general purpose" libraries; we don't use BioPython in my lab because we don't need 99% of it, and don't want to have to debug it.

In some sense, a lot of this is covered in Morgan Taschuk and Greg
Wilson's piece on making research software robust. Maybe the authors
could focus on what they DON'T cover?
Minor issues --
there's no guidance on choosing a license, at all. At the very least some
citations would probably be a good idea. And please mention OSI licensing;
there are a list of approved open source licenses to use, period.
The statement "use containers" is somewhat undermined by "sometimes you can't".

Methods

Are the methods appropriate to the aims of the study, are they well described, and are necessary controls included? Choose an item.

Conclusions

Are the conclusions adequately supported by the data shown? Choose an item.

Reporting Standards

Does the manuscript adhere to the journal's guidelines on [minimum standards of reporting?](#) Choose an item.

Choose an item.

Statistics

Are you able to assess all statistics in the manuscript, including the appropriateness of statistical tests used? Choose an item.

Quality of Written English

Please indicate the quality of language in the manuscript: Choose an item.

Declaration of Competing Interests

Please complete a declaration of competing interests, considering the following questions:

- Have you in the past five years received reimbursements, fees, funding, or salary from an organisation that may in any way gain or lose financially from the publication of this manuscript, either now or in the future?
- Do you hold any stocks or shares in an organisation that may in any way gain or lose financially from the publication of this manuscript, either now or in the future?
- Do you hold or are you currently applying for any patents relating to the content of the manuscript?

- Have you received reimbursements, fees, funding, or salary from an organization that holds or has applied for patents relating to the content of the manuscript?
- Do you have any other financial competing interests?
- Do you have any non-financial competing interests in relation to this paper?

If you can answer no to all of the above, write 'I declare that I have no competing interests' below. If your reply is yes to any, please give details below.

I declare that I have no competing interests.

I agree to the open peer review policy of the journal. I understand that my name will be included on my report to the authors and, if the manuscript is accepted for publication, my named report including any attachments I upload will be posted on the website along with the authors' responses. I agree for my report to be made available under an Open Access Creative Commons CC-BY license (http://creativecommons.org/licenses/by/4.0/). I understand that any comments which I do not wish to be included in my named report can be included as confidential comments to the editors, which will not be published.

I agree to the open peer review policy of the journal

To further support our reviewers, we have joined with Publons, where you can gain additional credit to further highlight your hard work (see: https://publons.com/journal/530/gigascience). On publication of this paper, your review will be automatically added to Publons, you can then choose whether or not to claim your Publons credit. I understand this statement.

Yes Choose an item.
