# Supplementary material for: Software engineering for scientific big data analysis
Source: Gigascience. 2019 May 23;8(5):giz054. doi: 10.1093/gigascience/giz054 (PMC6532757; doi:10.1093/gigascience/giz054)
Supplement: giz054_Reviewer_2_Report_Revision_1 -- C. Titus Brown — 2/5/2019 Reviewed [file giz054_reviewer_2_report_revision_1.pdf]

Reviewer Report

Title: Software engineering for scientific big data analysis

Version: Revision 1 **Date:** 2/5/2019

Reviewer name: C. Titus Brown

Reviewer Comments to Author:

In the manuscript "Software engineering for scientific big data analysis", the authors lay out a set of rules or guidelines for developing software to aid in, well, big data analysis!
This is revision 1 of the paper.
While I don't entirely agree with the detailed recommendations and could argue strongly about a few of them, that's ok - I think the paper makes a number of valuable recommendations for the field.
In particular, the authors have a lot of experience with workflow systems, and their recommendations there mirror my own realizations (which cost me about a decade of software development), so I hope they can get out there!
In the response to reviewers, the authors seem to narrowly address the concern that both reviewers had about the "big design up front" approach suggested in the paper. On reading the revisions, I think the reviewers did a reasonable job of addressing this concern. There are several places where I suggest some modifications or softening of perspective (below), but overall this is a very nice paper.
---
p3 line 56, "and it should be converged upon" - remove "it"
p4 line 22-25, suggest modifying this to avoid the requirement of upfront design: "and mindful planning must be engaged in _while_ developing computational tools." then remove "Before a single line of code is written..." The paragraph reads just as well, is less prescriptive, and addresses both reviewers concerns better!
p5 line 40, here I would note that the professional software industry is quite bad at determing whether adoption, refactoring, or rewriting is the best approach. It seems odd to argue that scientists should be better than industry here.
p6, line 17: I can think of many situations in which known-good outputs are virtually impossible to develop, e.g. quite a bit of bioinformatics research software where approximations and details are unknown until the analysis is done. I don't want to get too philosophical here, but I might argue that the point of new Big Data software is to do analysis

jobs that are too big for current software. This leaves the developer following this recommendation in the odd position of having to develop known good outputs for simple inputs, which then don't really apply to the interesting behavior of the program. Good practice, yes (we do this) but not prescriptive and certainly not complete.
(The whole of guideline 2 excludes most existing software in science! I'm not sure I disagree, but it's a pretty high bar.)
---
p7 line 29 - why is breaking backwards compatibility bad? Python does it regularly (if slowly). The key (in my opinion) is to be aware of it and deal with it, perhaps through semantic versioning, not to avoid it at all costs.
I would argue that testing facilitates refactoring, and that's a real point of guideline 2 and 3 - you want to be able to change your software without breaking it. Unchanging software is dead.

Methods

Are the methods appropriate to the aims of the study, are they well described, and are necessary controls included? Choose an item.

Conclusions

Are the conclusions adequately supported by the data shown? Choose an item.

Reporting Standards

Does the manuscript adhere to the journal's guidelines on [minimum standards of reporting?] Choose an item.

Choose an item.

Statistics

Are you able to assess all statistics in the manuscript, including the appropriateness of statistical tests used? Choose an item.

Quality of Written English

Please indicate the quality of language in the manuscript: Choose an item.

Declaration of Competing Interests

Please complete a declaration of competing interests, considering the following questions:

- Have you in the past five years received reimbursements, fees, funding, or salary from an organisation that may in any way gain or lose financially from the publication of this manuscript, either now or in the future?
- Do you hold any stocks or shares in an organisation that may in any way gain or lose financially from the publication of this manuscript, either now or in the future?
- Do you hold or are you currently applying for any patents relating to the content of the manuscript?
- Have you received reimbursements, fees, funding, or salary from an organization that holds or has applied for patents relating to the content of the manuscript?
- Do you have any other financial competing interests?
- Do you have any non-financial competing interests in relation to this paper?

If you can answer no to all of the above, write 'I declare that I have no competing interests' below. If your reply is yes to any, please give details below.

I declare that I have no competing interests.

I agree to the open peer review policy of the journal. I understand that my name will be included on my report to the authors and, if the manuscript is accepted for publication, my named report including any attachments I upload will be posted on the website along with the authors' responses. I agree for my report to be made available under an Open Access Creative Commons CC-BY license (http://creativecommons.org/licenses/by/4.0/). I understand that any comments which I do not wish to be included in my named report can be included as confidential comments to the editors, which will not be published.

I agree to the open peer review policy of the journal

To further support our reviewers, we have joined with Publons, where you can gain additional credit to further highlight your hard work (see: https://publons.com/journal/530/gigascience). On publication of this paper, your review will be automatically added to Publons, you can then choose whether or not to claim your Publons credit. I understand this statement.

Yes Choose an item.
